# Editorial: The expanding spectrum of hemophagocytic lymphohistiocytosis: pathogenic mechanisms and therapeutic implications

**DOI:** 10.3389/fimmu.2026.2017167

**Published:** 2026-10-21

**Authors:** Tiziana Lorenzini, Ola Sabet, Yasmine El Chazli, Maarja Soomann, Jana Pachlopnik Schmid

**Affiliations:** 1 Division of Immunology and the Children’s Research Center, University Children’s Hospital Zurich, Zurich, Switzerland; 2 Pediatric Immunology, Faculty of Medicine, University of Zurich, Zurich, Switzerland; 3 Children’s Cancer Hospital Egypt 57357, Cairo, Egypt; 4 Pediatric Hematology/Oncology Unit, Department of Pediatrics, Faculty of Medicine, Alexandria University, Alexandria, Egypt

**Keywords:** adult, cytotoxic lymphocytes, hemophagocytic lymphohistiocytosis, inborn errors of immunity, infections, macrophage activation syndrome, malignancies, pediatric

Hemophagocytic Lymphohistiocytosis (HLH) is a life-threatening syndrome characterized by clinical manifestations and laboratory abnormalities reflecting systemic hyperinflammation, together with immunological features of uncontrolled activation and tissue infiltration of T cells and macrophages (1). Historically, HLH has been classified as primary when caused by inherited defects in the effector molecules and pathways required for granule-dependent cytotoxicity of cytotoxic lymphocytes (1), and as secondary when associated with predisposing conditions such as unresolved infections, malignancies, autoimmune/autoinflammatory diseases, and medications (2). Emerging evidence suggests that, rather than representing a dichotomy, HLH encompasses a broad spectrum of disorders the share converging pathways but also exhibit distinctive pathogenic mechanisms, potentially informing diagnostic, monitoring, and therapeutic strategies (3). This collection illustrates how pediatric and adult HLH are embedded within this spectrum, with overlapping yet divergent profiles.

Beyond classic cytotoxic defects underlying familial HLH, resulting from biallelic pathogenic variants in *PRF1*, *UNC13D*, *STX11*, *STXBP2*, *RAB27A*, *LYST*, and *AP3B1* (1), an increasing number of inborn errors of immunity have been recognized as monogenic disorders predisposing to HLH. One example is NBAS deficiency, which has also been associated with impaired NK cell cytotoxicity; however, this dysfunction can be rescued by interleukin (IL)-2-induced NK cell activation, and the HLH phenotype is not invariably observed (4). Gong et al. described a novel compound heterozygous *NBAS* variant in a patient who developed progressive, fatal HLH. Based on colocalization experiments in mutant cells, they proposed that altered vesicular endoplasmic reticulum (ER)-Golgi trafficking might contribute to HLH susceptibility. Whether this leads to ER stress and inflammatory amplification remains to be established. These observations broaden the pathogenic framework of HLH beyond defects in cytotoxic granule release alone, implicating disturbances in intracellular trafficking and cellular stress responses as potential contributors to immune dysregulation. This proposed link is of interest in light of findings in patients with *CD48* variants and HLH-like disease, in whom impaired CD48 surface localization was accompanied by ER stress signaling and T cell dysfunction (5).

A second example is provided by Yu et al., who reported two cases of combined immunodeficiency due to heterozygous *ITPR3* variants associated with Epstein-Barr virus (EBV)-induced HLH. A combination of impaired lymphocyte activation, compromised control of EBV infection, and defective cytotoxicity may underlie the predisposition to HLH in these patients. These cases illustrate that susceptibility to HLH may arise from immune signaling defects that impair antiviral control.

Infections may act as triggers in patients with known genetic defects, but may also predispose to HLH in patients in whom an underlying genetic susceptibility has not been identified. This is illustrated by Liu et al., who investigated a large cohort of children with genetically undetermined HLH and found that infections, particularly EBV, were the most common associated conditions, whereas rheumatic and malignant diseases were less frequent.

Freeman et al. described two pediatric cases of systemic juvenile idiopathic arthritis (sJIA) complicated by lung disease and macrophage activation syndrome (MAS). Given the rarity and severity of these complications in children, treatment strategies are not yet fully standardized. The authors reported successful outcomes in two cases of refractory sJIA-associated lung disease treated with cyclophosphamide and rituximab. The clinical overlap between MAS and HLH, together with the observation that MAS, although most commonly associated with rheumatic diseases, can also arise in the context of an underlying genetic defect (6), further underscores that the distinction between primary and secondary HLH is not absolute.

Compared with pediatric HLH, adult HLH is less frequently attributable to underlying genetic defects and more often occurs in the context of complex acquired conditions. This has raised questions about the applicability of the HLH-2004 criteria and the HLH-94 treatment protocol to adults (7), given that they were originally developed in pediatric HLH cohorts. Revised diagnostic guidelines for familial HLH were published in 2024 (8); however, they do not constitute a general update of the HLH-2004 criteria for adult HLH. The HScore, which integrates clinical, biological, and cytological features, has been introduced to estimate the probability of HLH in adults (9).

Importantly, a genetic etiology can still be identified in some adults presenting with HLH (10). Wei et al. reported two patients with adult-onset familial HLH due to compound heterozygous hypomorphic *UNC13D* variants. Interestingly, in both patients, peripheral neuropathy preceded the onset of HLH. They hypothesized that impaired lymphocyte cytotoxicity may promote localized, subclinical immune activation prior to the development of overt multisystemic inflammation. This temporal dissociation suggests that inherited defects predisposing to HLH may initially manifest as organ-specific or atypical immune-mediated phenotypes before the emergence of the full systemic syndrome.

However, in adults, malignancies are the most frequent conditions associated with HLH, followed by infections, as documented by Xie et al. in a large patient cohort. They observed that malignancy-associated HLH was associated with lower survival, potentially reflecting the lack of standardized and specific treatment strategies. Haar et al. analyzed an adult cohort of patients with HLH admitted to the intensive care unit and documented a similar pattern, with malignancies being the most frequent associated condition and carrying relatively high mortality. They additionally noted that, although nearly all critically ill patients met the HLH-2004 criteria and exceeded the diagnostic HScore threshold, differentiating HLH from sepsis and multi-organ failure remained challenging. They also highlighted the need for reliable prognostic markers.

Huang et al. described an adult patient with extranodal NK/T-cell lymphoma who developed refractory HLH progression despite achieving a radiologic complete metabolic response following standard chemotherapy. They suggested that serial assessment of immune activation and HLH disease activity biomarkers should complement tumor burden monitoring to facilitate early identification and treatment of malignancy-associated HLH. This clinical dissociation suggests that control of the underlying malignancy does not necessarily lead to resolution of the hyperinflammatory program once HLH has been established.

In conclusion, further research is needed to fully elucidate the etiologies, pathogenic mechanisms, diagnostic biomarkers, and treatment approaches in HLH. Defining the common pathways that converge across the spectrum of pediatric and adult HLH may help identify shared therapeutic strategies, while recognizing disease-specific differences may improve risk stratification, monitoring, and treatment selection.
